# Interpretation of Reflection and Colorimetry Characteristics of Indium-Particle Films by Means of Ellipsometric Modeling

**DOI:** 10.3390/nano13030383

**Published:** 2023-01-18

**Authors:** Hao-Tian Zhang, Rong He, Lei Peng, Yu-Ting Yang, Xiao-Jie Sun, Yu-Shan Zhang, Yu-Xiang Zheng, Bao-Jian Liu, Rong-Jun Zhang, Song-You Wang, Jing Li, Young-Pak Lee, Liang-Yao Chen

**Affiliations:** 1 Department of Optical Science and Engineering, School of Information Science and Technology, Fudan University, Shanghai 200433, China; 2 High Tech Center for New Materials, Novel Devices and Cutting-Edge Manufacturing, Yiwu Research Institute, Fudan University, Yiwu 322000, China; 3 Shanghai Institute of Technical Physics, Chinese Academy of Sciences, Shanghai 200083, China; 4 Department of Physics, Quantum Photonic Science Research Center and RINS, Hanyang University, Seoul 04763, Republic of Korea

**Keywords:** indium-particle film, reflectance spectrum, spectroscopic ellipsometry, oscillator decomposition

## Abstract

It is of great technological importance in the field of plasmonic color generation to establish and understand the relationship between optical responses and the reflectance of metallic nanoparticles. Previously, a series of indium nanoparticle ensembles were fabricated using electron beam evaporation and inspected using spectroscopic ellipsometry (SE). The multi-oscillator Lorentz–Drude model demonstrated the optical responses of indium nanoparticles with different sizes and size distributions. The reflectance spectra and colorimetry characteristics of indium nanoparticles with unimodal and bimodal size distributions were interpreted based on the SE analysis. The trends of reflectance spectra were explained by the transfer matrix method. The effects of optical constants *n* and *k* of indium on the reflectance were demonstrated by mapping the reflectance contour lines on the *n*-*k* plane. Using oscillator decomposition, the influence of different electron behaviors in various indium structures on the reflectance spectra was revealed intuitively. The contribution of each oscillator on the colorimetry characteristics, including hue, lightness and saturation, were determined and discussed from the reflectance spectral analysis.

## 1. Introduction

Indium is a plasmonic metal that has attracted considerable attention for decades [1,2,3,4,5,6,7,8,9,10,11,12,13]. Indium does not have interband transitions in the visible range like gold and silver [1,2]. Therefore, indium can support the plasmonic resonances in the ultraviolet (UV) band [3]. Indium is widely used in plasmonics because its localized surface plasmon resonance (LSPR) can be regulated by its structural parameters [4,5], the interaction with the substrate [6] and the oxide layer [7]. As one of the most commonly used structures, particles can be easily prepared and modified. For example, indium-particle films have shown superiority in UV-excited surface-enhanced Raman scattering [8,9,10], UV fluorescence enhancement [11] and solar absorption [12,13]. At the same time, the unique spectral response characteristics of metal particles promote their use in the field of structural colors [14,15]. Several UV plasmonic metals were investigated for structural coloring [16,17]. To facilitate the nanostructures with color vibrancy and angular independence, etc., it is crucial to understand and regulate the reflectance spectra of metal structures. As one of the advantageous plasmonic materials, the structure-dependent reflection properties of indium particles deserve further exploration.

The geometry and optical properties of the material determine the reflectance spectrum of a nanostructure. Therefore, to understand the reflection spectral properties, it is necessary to obtain both optical constants and structural information about the material. Ellipsometry is a powerful tool to characterize materials’ structural information and optical properties [18]. On the basis of appropriate optical and dispersion models, we can infer the optical constants and structural information of the examined sample from the ratio of complex reflection coefficients measured by ellipsometry. Ellipsometry is advantageous in analyzing complex structures [19,20,21]. Moreover, researchers can monitor the growth of a metal sample in real-time through ellipsometry to obtain its structural and optical properties [22]. For metal particles growing in size, the evolution of their optical constants can be also obtained directly by ellipsometry [4,6,23]. From this perspective, ellipsometry provides a solid foundation for understanding the reflection spectrum and the powerful chromatic performance of metal structures.

Physical vapor deposition techniques, such as thermal evaporation, molecular-beam epitaxy, electron-beam evaporation, etc., are often employed in preparing non-sequential particle films, owing to the simple process without subsequent processing [16,23,24]. However, the prepared particle film presents a certain particle distribution, even a bimodal particle distribution [23,24]. The size distribution of particles affects the damping factor of resonance [4], and a bimodal size distribution might result in the emergence of multiple resonance peaks [23,24]. For samples with a bimodal particle size distribution, ellipsometry can directly model and distinguish the contributions of complicated structures to the overall response [24]. Furthermore, percolation is expected as the particles grow in size, which can be determined simply by measuring the ellipsometric parameters [25]. The ellipsometric modeling extracts the effect on the optical responses [22,24,25]. Such structure-dependent optical responses influence the reflectance spectrum consequently. To obtain accurately the structure-dependent optical response and establish its relationship with the reflectance spectrum is of great significance in revealing the underlying origins of a reflectance spectrum, thus providing the guidance to regulate the reflectance spectrum of particulate films and color rendering.

In this work, we analyze the reflection and colorimetry behavior of indium-particle films with unimodal and bimodal distributions based on our previous work [24]. The fitted optical constants and thicknesses reproduce well the reflectance spectra for all samples through the transfer-matrix method. The effective optical admittance of film and substrate interprets the dispersion of each reflectance spectrum. Furthermore, they are discussed in detail: the effects of different electron behaviors in large and small particles, and the percolated zones, as modeled by other Lorentz oscillators, on the reflectance spectra. By the vector decomposition, the contribution of each oscillator on the reflectance of indium film is illustrated clearly. From the reflectance spectra, we further derive the colorimetric parameters such as each sample’s hue, lightness and saturation. The structure-originated changes of these parameters are discussed. We believe that the ellipsometry-assisted exploitation of the structure-dependent reflectance of complicated metallic structures will provide an impetus to applying particulate indium films in a plasmonic color generation.

## 2. Materials and Methods

**Figure 1:**
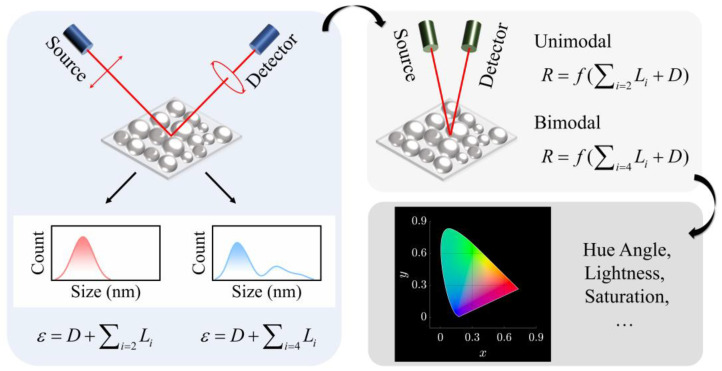
Flowchart of the ellipsometry-assisted interpretation on reflection and colorimetry of nanosized indium particles.

Figure 1 is the flowchart of this work. Six indium-particle films were deposited on silicon (Si) substrates using electron beam evaporation under a vacuum level of 2 × 10^−2^ Pa. The sizes and distributions of particulate indium were controlled by varying the deposition time. With increasing deposition time, the particle size of indium grows continuously, and the size distribution evolves from unimodal to bimodal. The prepared samples’ optical responses in 200–1000 nm, labeled by S1–S6, were investigated using a spectroscopic ellipsometer (M2000X-FB-300XTF, J. A. Woollam Inc., Shanghai, China) in advance. In the fitting process, the films with unimodal size-distributions (S1, S2 and S3) and those with bimodal size distributions (S4, S5 and S6) were modeled using different sets of oscillators. For S1–S3, two Lorentz oscillators, L1 and L2, were used, representing the comprehensive contributions from the high-energy range and the LSPR in indium particles, respectively. For S4–S6, two other oscillators, L3 and L4, were used, describing the LSPR in small indium particles and the electron delocalization in percolated zones, respectively. The ellipsometric investigation and modeling of indium samples are briefly shown in the left box in Figure 1. More details about the sample preparation and characterization were presented in our previous work [24]. As displayed in the right-upper space of Figure 1, the reflectance consists of parts, resulting from different electron behaviors. In the ellipsometric modeling, those electron behaviors were described by the Drude term or Lorentz oscillators. According to the ellipsometric modeling, we reveal the physical origins of the reflection behaviors of samples. Finally, the reflectance spectra are converted into color coordinates in the CIELAB color space [26]. The analysis of hue, lightness and saturation can be performed subsequently. The interpretation of colorimetry parameters gives information not only on the appearance of particle films, but also on the underlying reasons for it [27,28].

## 3. Results and Discussion

The reflectance spectra of S1–S6 can be derived from the ellipsometric modeling to reproduce well the measured ones. For S1–S4, the reflectance increases first, reaches the maximum value of around 0.40, and does not vary significantly after it [24]. We define a knee point where the reflectance goes to be flat, as observed in the spectra of S1, S2, S3 and S4. The knee point of S1–S4 red-shifts continuously, as shown in our previous work (Figure 7 in Ref. [24]). For S5 and S6, the reflectance increases monotonically in the measured spectral range and no knee point exists [24]. We conduct a two-step analysis to interpret the reflection behavior of the indium particles. The colorimetric parameters are deduced and discussed subsequently.

### 3.1. Effective Structure with a Single Interface

First of all, we treat the indium film and Si substrate as an effective semi-infinite layer. Therefore, we simplify the three-layer model (air/indium film/Si substrate) used in the ellipsometric analysis and reduce the number of interfaces to one (air/effective layer). In this case, the reflectance *R* is governed by the index contrast between air and the effective layer and is expressed as [29].

(1) $$R=\left(\frac{Y-Y_{0}}{Y+Y_{0}}\right){\left(\frac{Y-Y_{0}}{Y+Y_{0}}\right)}^{*},$$

where *Y* is the optical admittance of the effective layer, which is numerically equal to the complex refractive index *n*_eff_ + i*k*_eff_, determined by using the transfer-matrix method [30]. *Y*_0_ is the optical admittance of air. Furthermore, we can analytically de rive the following relation,

(2) $${\left(n_{\mathrm{eff}}+\frac{R+1}{R-1}\right)}^{2}+k_{\mathrm{eff}}{}^{2}={\left(\frac{R+1}{R-1}\right)}^{2}-1.$$

**Figure 2:**
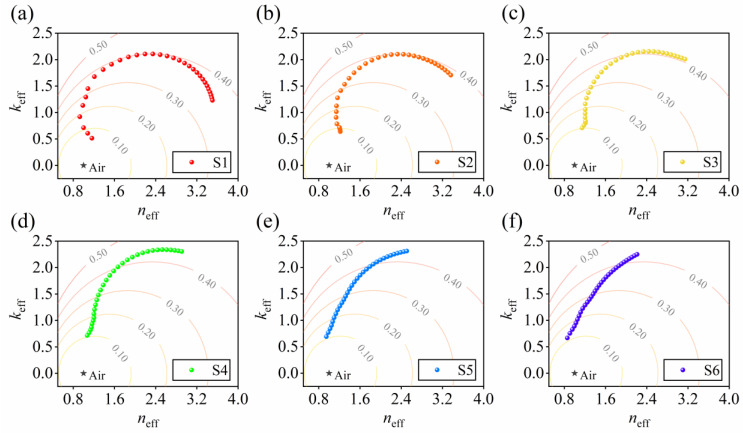
Trajectories of the optical admittance of (**a**) S1, (**b**) S2, (**c**) S3, (**d**) S4, (**e**) S5 and (**f**) S6 in 210–1000 nm. Points near the origin correspond to short wavelengths. Light lines represent the contour lines of reflectance. The black star indicates the admittance of air.

Equation (2) is in the form of a circle function when *R* is a constant, meaning that the contour lines of reflectance are a series of circles in the *n*_eff_-*k*_eff_ plane. Figure 2a–f plot *n*_eff_ and *k*_eff_ of S1–S6 in 210–1000 nm and depict the contour lines of reflectance at levels of 0.10, 0.20, 0.30, 0.40 and 0.50. Dots near the origin correspond to short wavelengths. Light lines indicate the contour lines of reflectance. It is intuitive that, for S1–S4, *k*_eff_ goes to be larger first, and decreases at longer wavelengths. The reflectance increases at the beginning and comes to be flat after *k*_eff_ drops, as the normal of the *n*_eff_-*k*_eff_ trajectory reaches the normal of the neighboring contour line. For S5 and S6, no peak of *k*_eff_ exists. Both *n*_eff_ and *k*_eff_ increase at longer wavelengths. The trajectories of *n*_eff_-*k*_eff_ continuously cross the contour lines. Consequently, the reflectance of S5 and S6 increases monotonically. The combination of the *n*_eff_-*k*_eff_ plot and contour lines of reflectance offers a simple and intuitive way to interpret the dispersion trends of the reflectance spectra.

**Figure 3:**
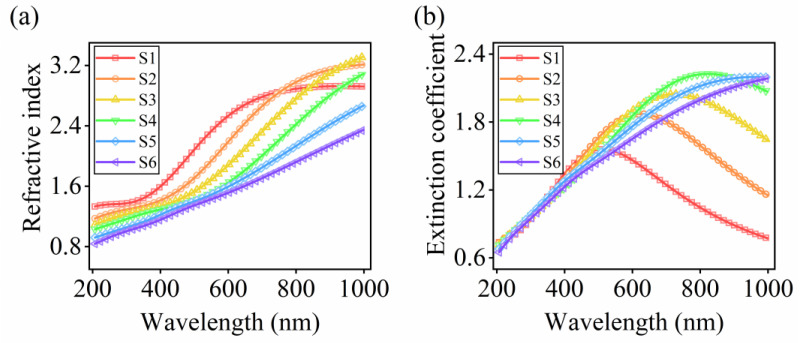
(**a**) Refractive index and (**b**) extinction coefficient of indium-particle films S1–S6.

As the effective layer is composed of of Si and indium film, the effective optical constants are affected by the substrate. To eliminate the influence from the substrate, we depict the spectra of refractive indices *n* and extinction coefficients *k* of six indium-particle films, as shown in Figure 3. The refractive index *n* presents the abnormal dispersion and shows a redshift from S1 to S6. The extinction coefficient *k* exhibits a red-shifting peak for S1–S4. For thicker films, S5 and S6, no *k* peak exists. The redshifts of *n* and *k* of particle films contribute to the redshift of knee points in the reflectance spectra. The noticeable redshift of the optical constant results from that of the plasmon resonance. For finite-sized particles, an increase in particle size shifts the Frohlich frequency to lower values, according to the Mie theory [31,32]. In addition, when the incident electric field travels across the entire metal structure, the induced dynamic depolarization field also leads to the movement of the LSPR peak [33]. In addition, other electron behaviors, resulting from different structures inside the indium films, also affect the dielectric responses of particulate indium films, and thereby influence the reflectance spectra [24]. Therefore, in the second step, we seek to exploit the contributions of different electron behaviors on the reflection based on the ellipsometric analysis.

### 3.2. Effects of n, k of Indium Films on the Reflectance Spectra

In this step, we return to the three-layer model (air/indium film/Si substrate). The reflectance of a single-film structure is determined by the interference of reflected light from two interfaces, which is expressed as [34].

(3) $$R={\left|\frac{r_{01}+r_{12}\exp(i2\delta )}{1+r_{01}r_{12}\exp(i2\delta )}\right|}^{2},$$

where *r*_01_ and *r*_12_ denote the reflection coefficients at the air/film interface and the film/substrate interface, respectively. *δ* = 2π*Nd*cos*θ/λ* is the phase in the film, with *N = n +* i*k* being the complex refractive index, *d* the thickness of indium film, *λ* the wavelength and *θ* the angle of incidence at the air/film interface. As *r*_01_ and *r*_12_ are determined by the contrast of optical admittances of the media, the reflectance spectra are parameterized using *n*, *k* and *d* of the indium films at a known angle of incidence. To illustrate the influences of optical constants, *n* and *k*, on the reflectance spectra, we can depict the evolution of reflectance spectra for S1–S6 by increasing the number of Lorentz oscillator components. Furthermore, we can link the optical constants of each indium film to the reflectance by calculating the contour lines of reflectance directly in the *n*-*k* plane given a specific wavelength and sample thickness. The corresponding *n*-*k* pair of an indium film at the wavelength is marked in the plane. By using the oscillator decomposition, the location of an *n*-*k* pair is the sum of a series of vectors, where each vector represents the changes of *n* and *k* induced by the newly added oscillator. By mapping the vectors, we can obtain the effect of each oscillator, i.e., different electron behaviors, revealed by the ellipsometric analysis, on the reflectance of indium-particle films.

#### 3.2.1. Oscillator Decomposition for the Particulate Indium with a Unimodal Distribution

**Figure 4:**
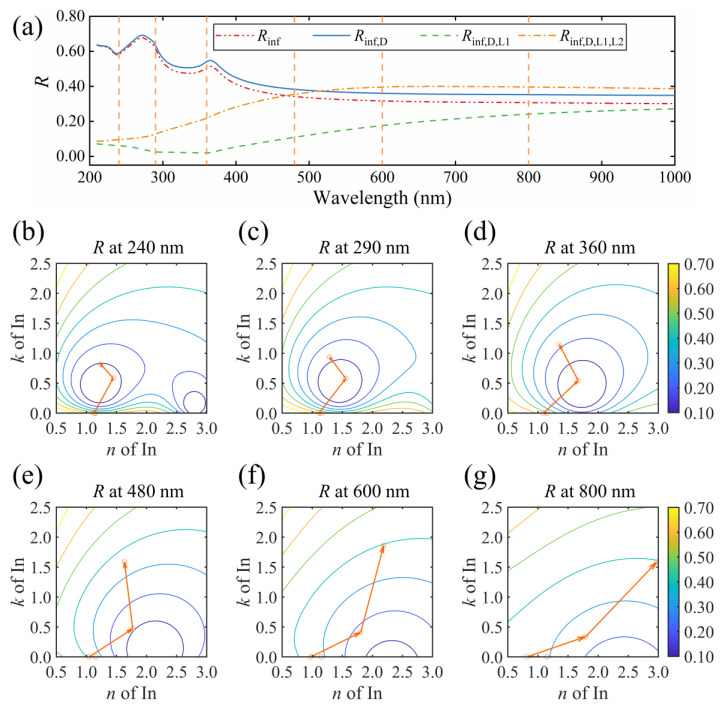
Reflectance spectra with different Lorentz oscillator components for (**a**) S2, and the oscillator decomposition at a wavelength of (**b**) 240, (**c**) 290, (**d**) 360, (**e**) 480, (**f**) 600 and (**g**) 800 nm. Curves in (**b**–**g**) are the contour lines of reflectance.

The reflectance evolution and vector decomposition at representative wavelengths for S2 are shown in Figure 4. In Figure 4a, subscripts inf, D, L1 and L2 represent the high-frequency dielectric constant, Drude term, high-frequency contribution and LSPR, respectively. These four parts contribute to the entire dielectric response of particulate indium with unimodal distribution [24]. To investigate the effect of each component, the order was determined first. The high-frequency dielectric term is a constant, describing the dielectric response out of the measured range. We treat it as the first term. The Drude term is dispersed weakly in the measured range, with a negligible contribution to the absorption. We thereby put it ahead of the Lorentz oscillators. In terms of the Lorentz oscillators, the contribution in the high-frequency range serves as a background for the absorption, while the LSPR is the dominant behavior of indium particles upon the incident light [4,6,9,24]. Therefore, we set the LSPR of indium particles as the last component. In Figure 4a, when the dielectric function of S2 comprises solely the high-frequency dielectric constant, the reflectance spectrum *R*_inf_ is similar to that of the air/Si structure since there is no dispersion in the film. By adding the Drude term, the *R*_inf,D_ slightly increases. When the high-energy contribution is considered, the *R*_inf,D,L1_ decreases dramatically in the short-wavelength region. Finally, the LSPR of indium particles enhances the reflectance in the entire spectral range and completes the final reflectance spectrum.

Figure 4b–g display the vector decompositions and contour lines of reflectance at selective wavelengths of 240, 290, 360, 480, 600 and 800 nm, respectively. At each wavelength, the starting point marks the high-frequency optical constant (*n*_inf_, *k*_inf_). The first vector points to (*n*_inf,D_, *k*_inf,D_) and describes the change induced by the Drude part of the indium film. At short wavelengths, such as 240, 290 and 360 nm, the Drude term shows little effect on the optical constant of the sample. At wavelengths of 480, 600 and 800 nm, the influence of the Drude term becomes noticeable. A reduction of *n* occurs while no change of *k* is observed. In our previous study, the intraband transition of free electrons hardly contributes to the absorption of indium films [24]. The susceptibility *χ*, described by using the Drude model, is reduced to be *χ* = −*ω*_p_^2^/*ω*^2^ [1,35,36,37], with *ω*_p_ being the plasma frequency and *ω* the light frequency. Addition of the negative Drude term results in a drop in *n* of the sample. Hence, the index contrast at the film/Si interface becomes more prominent, increasing the reflectance consequently.

The second vector points to (*n*_inf,D,L1_, *k*_inf,D,L1_) describe the comprehensive contribution from the high-energy range. This Lorentz oscillator introduces an increment in both *n* and *k*, as displayed in Figure 4b–g. In the spectrum of the dielectric function, this part serves as a baseline [24]. However, the change in the reflectance spectrum is drastic. At wavelengths of 240, 290 and 360 nm, the second vector continuously crosses the contour lines of reflectance, inducing a steep drop in the reflectance value. The reflectance at these wavelengths is determined by the reflectivity at each interface. For a bare Si substrate, the contrast between air and Si is large [38], leading to a strong reflection at the air/Si interface at these wavelengths. When a thin film with small *k* and *n* is inserted, the contrast at the air/film interface is relatively small. As a result, the reflection in the first interface is weak, and a large ratio of incident light was transmitted into and absorbed by the inserted film. As the contrast at the film/Si interface is large, a strong reflection is expected at this interface, and the reflected light is absorbed again in the film. In this process, the reflectance is suppressed by absorption. Since the absorption coefficient is expressed as *α* = 4 *k*π/*λ*, with *λ* representing the wavelength, the absorption is sensitive to *k* at short wavelengths [39].

Consequently, a change in *k* causes a notable drop in the reflectance. For instance, *k* of 0.2 at 240 nm attenuates approximately 44% of the reflection amplitude from the film/Si interface. As *k* grows continuously, the reflection at the air/film interface is enhanced. The competition between reflection at the air/film interface and film absorption leads to a local minimum of the reflectance, as shown in the contour lines in Figure 4b–d. In Figure 4e–g, the influence of *k* on *α* is less pronounced at longer wavelengths. The drop in reflectance is caused by the increase in *n*, which reduces the steep change of the refractive index from the air to Si.

Finally, the LSPR is considered, as the third vector shows. For S2, the resonance wavelength is around 544 nm, where the displacement reaches maximum amplitude and is strictly 90° out of phase with respect to the electric field [40]. At wavelengths of 240, 290, 360 and 480 nm, the electric susceptibility approaches the resonant phase condition, inducing a drop of *n* and an increment of *k* for indium. The reflectance increases continuously. At wavelengths of 600 and 800 nm, the L2 oscillator induces an increment of *n*, as the displacement comes to be in phase with the varying electric field.

Meanwhile, the *k* component of the third vector starts to drop. Therefore, the modulus of the third vector keeps nearly constant. The third vector is rotated in the *n*-*k* plane, and the endpoint of the vector coincides with the contour line, resulting in a flat reflectance spectrum in the long-wavelength region. In summary, the high-frequency dielectric constant and the Drude term induce only a slight change in reflectance. The high-energy contributions induce a steep drop in reflectance, which can be interpreted as the film absorption and reflection competition at the first interface. The LSPR enhances the reflectance, leading to the emergence of knee point in the reflectance spectrum as the endpoint of the third vector evolves along the contour line in the *n*-*k* plane.

#### 3.2.2. Oscillator Decomposition for the Particulate Indium with a Bimodal Distribution

**Figure 5:**
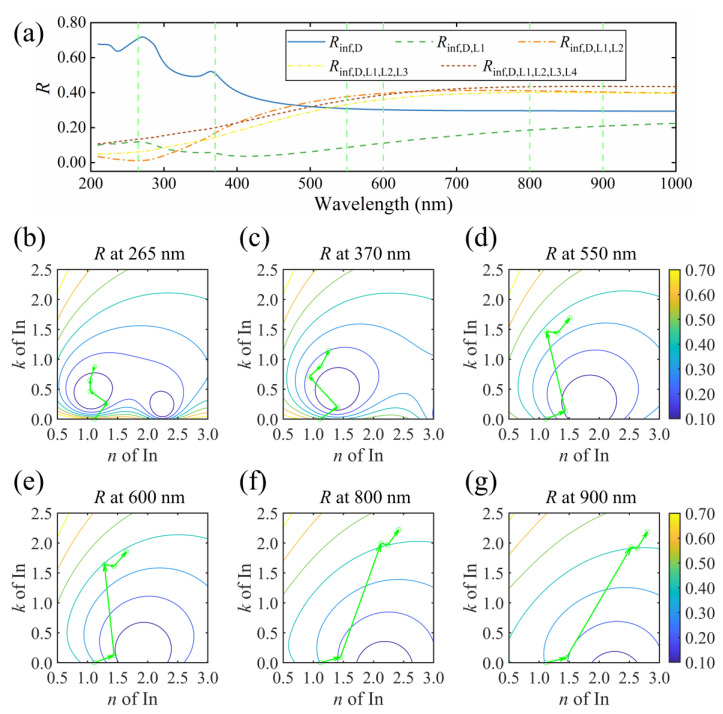
Reflectance spectra with different Lorentz oscillator components for (**a**) S4 and the oscillator decomposition at a wavelength of (**b**) 265, (**c**) 370, (**d**) 550, (**e**) 600, (**f**) 800 and (**g**) 900 nm. Curves in (**b**–**g**) are the contour lines of reflectance.

The evolution of the reflectance and vector decomposition for S4 are shown in Figure 5. Since the size distribution of S4 is bimodal, four oscillators were used to parameterize the optical responses. As the high-frequency dielectric constant and the Drude term yield little effect on the reflectance, we combine these two parts, marked as *R*_inf,D_ in Figure 5a. In addition, subscripts L1, L2, L3 and L4 represent the high-frequency contribution, the LSPR in large particles, the LSPR in small ones and the contribution of delocalized electrons in the percolated zones, respectively. In Figure 5a, L1 still leads to a drop over the entire spectrum. L2 results in an increment in the long-wavelength region. In the short-wavelength region, this induces a reduction of the reflectance. L3 enhances the reflection in the short-wavelength range; on the other hand, it slightly suppresses the reflection in the long-wavelength region. Finally, L4 leads to an increment over the entire reflectance spectrum of S4.

Figure 5b–g display the vector decomposition at wavelengths 265, 370, 550, 600, 800 and 900 nm. The vector decomposition starts from the sum of the high-frequency dielectric constant and the Drude term. The first vector describes the changes of *n* and *k* induced by the addition of L1, which behaves similarly to that of S2, as discussed above. The second vector describes the contribution of LSPR for large particles. At 265 nm, the LSPR adds a negative *n* and a small positive *k* to the film, reducing reflectance. At longer wavelengths, the addition of the LSPR part gives rise to the reflectance until the endpoint of the second vector starts to move along the contour line.

The third vector describes the contribution of LSPR for small particles, which is smaller in amplitude than the second vector. The resonance wavelength of L3 is 342 nm. Hence, the length of this vector is longer at short wavelengths. As the wavelength increases, this vector induces a negative change of *k*. Since the extinction of light corresponds to the polarization of matter upon the electromagnetic field, we address the negative growth of *k* from the relationship between *k* and dielectric function [39]. At long wavelengths such as 600, 800 and 900 nm, the material polarization comes to be in phase with the incident light, leading to a large real dielectric function *ε*_1_^L3^ of the LSPR for the small particles. The imaginary part *ε*_2_^L3^ is small, as the wavelength is away from the resonance. The ratio of the total imaginary part dielectric function *ε*_2_, to the total real part dielectric function *ε*_1_, is reduced by adding the oscillator describing the LSPR for the small particles. As $k={\left(\left(\sqrt{1+\varepsilon_{2}{}^{2}/\varepsilon_{1}{}^{2}}-1\right)/2\varepsilon_{1}\right)}^{1/2}$, a reduced *ε*_2_/*ε*_1_ and a large *ε*_1_ in the spectral region of long wavelength lead to a reduced *k* accordingly. Therefore, as the *k* component of the third vector changes from positive to negative, *R*_inf,D,L1,L2,L3_ increases at short wavelengths and decreases at longer wavelengths, as shown in Figure 5a.

Finally, the oscillator L4 describes the delocalization of electrons with a center-energy value close to zero. The metal-like behavior causes a strong light reflection, owing to the skin effect [41], thus giving rise to the reflectance at all wavelengths, as L4 induces increments of both *n* and *k*. Note that the increment in reflectance, induced by L4, is dispersed. As L4 centers at a low energy value, the change of *n* and *k* in the long-wavelength region is notable, consequently resulting in a change in reflectance. Meanwhile, the fourth vector crosses the contour lines nearly vertically at short wavelengths, which also induces a noticeable change in the reflectance. Therefore, the change in the reflectance spectrum, resulting from L4, reaches the minimum value in the midrange.

In the particle film with a bimodal size distribution, the addition of two oscillators, L3 and L4, also contributes to the red shift of the knee point in the reflectance spectrum. As shown in Figure 5e–g, the endpoints of the second vector locate in the neighborhood of the contour line. By adding two vectors, the endpoint for 600 nm (Figure 5e) stays near the same contour line. On the contrary, the endpoints for 800 and 900 nm (Figure 5f–g) move away from the former contour line, resulting in the reflectance increment between 600 and 800 nm, where the knee point of S4 lies inside. The sequence of L2, L3 and L4 can be changed during the decomposition, but the influence of each component on the reflectance does not vary significantly, as depicted in Figure S1 in Supplementary Materials.

#### 3.2.3. Oscillator Decomposition for the Particulate Indium without Reflectance Knee Point

**Figure 6:**
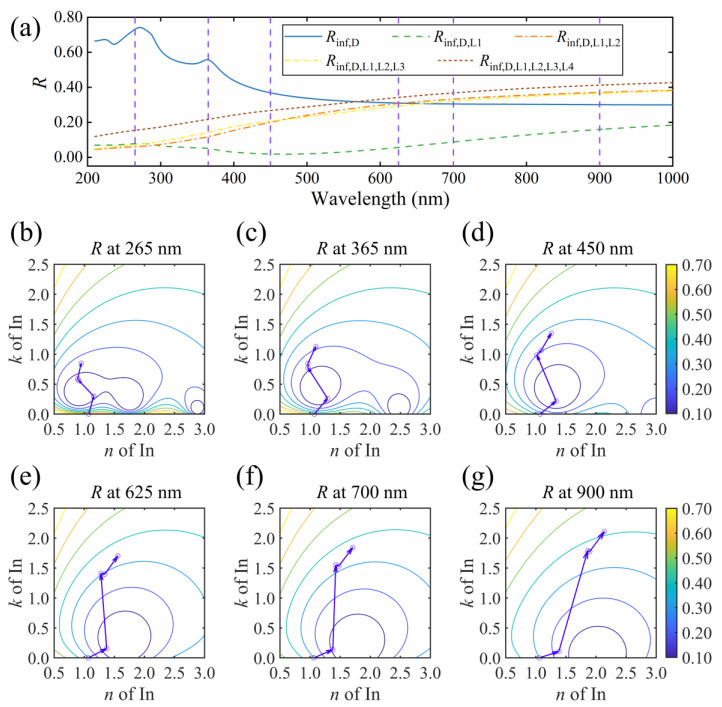
Reflectance spectra with different Lorentz oscillator components for (**a**) S6, and oscillator decomposition at a wavelength of (**b**) 265, (**c**) 365, (**d**) 450, (**e**) 625, (**f**) 700 and (**g**) 900 nm. Curves in (**b**–**g**) are the contour lines of reflectance.

Finally, in Figure 6, we depict the reflectance of S6, a monotonically increasing spectrum for the film containing both small and large particles and percolated zones. As shown in Figure 6a, similarly to S4, the third oscillator, L3 has different effects on the reflectance in different wavelength ranges. The addition of L4 increases the reflectance in the entire spectrum. The vector decompositions at 265, 365, 450, 625, 700 and 900 nm are illustrated in Figure 6b–g, respectively. By adding the first oscillator, the reflectance becomes smaller. By adding L2, both *n* and *k* of S6 present an abnormal dispersion in the entire spectral range, resulting in a monotonically increased reflectance spectrum. Therefore, the spectrum presents no knee point. L3 centers at 454 nm. At 265, 365 and 450 nm, the *k* component of vector L3 is positive, as displayed in Figure 6b–d. At longer wavelengths, vector L3 points to a negative *k* and thereby suppresses the reflectance, as discussed in the cases of S4. The effects of L4 remain the same as in S4. For S1, S3 and S5, oscillator decompositions are shown in Supplementary Materials (Figures S2–S4).

### 3.3. Evolutions of the Colorimetric Parameters

**Figure 7:**
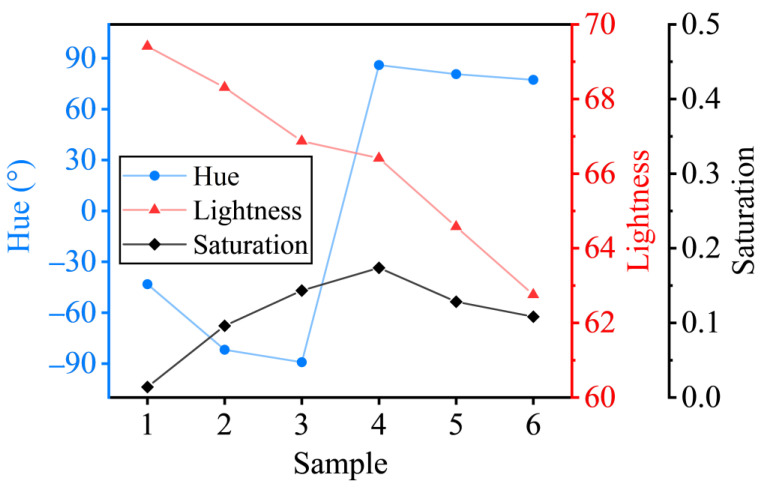
Hue, lightness and saturation of S1–S6.

Figure 7 depicts the evolution of colorimetric parameters, calculated from the reflectance, including hue angle, lightness L* and saturation *S*. The calculating procedure is shown in Supplementary Materials. A huge jump of the hue angle occurs between S3 and S4, where the additional two Lorentz oscillators are used in the ellipsometric modeling. The lightness falls for S1–S6. The saturation reaches the maximum and drops for S4–S6. Here, we focus on the different electron behaviors in different structures described by oscillators L2–L4. The effects of L2, L3 and L4 for S1–S6 are listed in Table 1. Parameter a* describes the appearance of magenta over the green, with negative a* to be green and positive a* to be magenta. Parameter b* describes the appearance of yellow over blue, with negative b* to be blue and positive b* to be yellow. For S1, the addition of L2 induces an increment in L* and a reduction in *S*. As the reflectance in the visible range is enhanced by L2; the lightness increases accordingly. The color saturation is reduced as the dispersion of reflectance is suppressed when L2 is considered. As the LSPR centers around 544 nm and causes an enhanced reflection at shorter wavelengths, parameters a* and b* decrease simultaneously. For S2 and S3, similar effects of L2 on L*, *S*, a* and b* are observed. From S1 to S3, since the particle size increases and the LSPR red-shifts, the reflection in the short-wavelength region is weakened consequently, which leads to a reduction of L*, and an increase of *S* and b*. Eventually, the hue angle is negative, approaching −90° as b* increases. For S4–S6, the added L3 oscillator reduces the reflectance in the short-wavelength region. In addition, the red-shifted L2 also renders the increment in a* from S4 to S6 when L1 and L2 are considered. These two LSPRs lead to an increased positive a* in S4–S6. The hue angle is shifted accordingly. For S4, L3 turns out to help enhance saturation. As discussed above, the enhancement of reflectance by L4 in short- and long-wavelength ranges is more notable than in the midrange. Consequently, L* and a* are increased, while *S* and b* are reduced when L4 is considered. For S5, L3 does not increase the saturation, while L4 suppresses it. Hence, the saturation of S5 starts to drop, compared with that of S4. For S6, the peak induced by L3 in the reflectance spectrum compensates the weak reflection region of L2. As a result, the saturation is suppressed furthermore. In summary, the hue angle jumps from negative to positive, as L2 and L3 contribute to the increment of coefficient a*. The lightness L* decreases monotonically, with the dominant LSPR for large particles red-shift from 544 nm to the infrared range. The saturation *S* first increases as the dispersion of reflectance become significant, while the addition of L3 in S6, as well as L4 in S4–S6, compensates for the low reflectance in the short-wavelength range.

## 4. Conclusions

The contributions of versatile electron behaviors to the reflection and colorimetry characteristics of indium-particle films in complicated structures are revealed from the ellipsometric modeling. The dispersions of reflectance spectra are explained by mapping the effective optical constants on the contour lines of reflectance. The effects of multiple oscillators are shown intuitively in vector form. The Drude term promotes slight reflectance. The contribution from the high-energy range reduces the reflectance as the index contrast is reduced, and the absorption enhances. The LSPRs for large and small particles are analyzed regarding the electric polarization in the representative wavelength ranges. The delocalization of electrons gives an increment in the whole range of the reflectance spectrum. The jump in the hue angle is caused by the red-shift of LSPR for large particles and the emergence of LSPR for small particles. The decreasing lightness results from the weakened reflection in the short-wavelength region. The emergence of L4, and the competition between L3 and red-shifted L1, render the maximization of saturation. This kind of incorporation with ellipsometry offers a strategy to understand and manipulate indium particles’ reflection and colorimetric behaviors, which is beneficial for the applications of indium-particle films in structural coloring.

## Figures and Tables

**Table 1:** Colorimetric parameters of S1–S6 with increasing the number of Lorentz oscillators.

Sample	Number of Oscillators	L*	a*	b*	Hue (°)	*S*
S1	1	59.35	0.08	3.41	88.69	0.06
	1, 2	69.41	−0.69	0.65	−43.30	0.01
S2	1	46.06	1.87	16.60	83.59	0.36
	1, 2	68.36	−0.92	6.54	−82.00	0.10
S3	1	31.61	5.65	15.53	70.00	0.52
	1, 2	66.91	−0.11	9.74	−89.34	0.15
S4	1	35.97	4.70	18.38	75.67	0.53
	1, 2	67.74	−0.70	10.26	−86.11	0.15
	1, 2, 3	64.32	0.39	13.72	88.38	0.21
	1, 2, 3, 4	66.41	0.82	11.50	85.90	0.17
S5	1	28.92	6.00	21.35	74.30	0.77
	1, 2	61.88	0.24	10.88	88.72	0.18
	1, 2, 3	60.55	1.21	10.76	83.59	0.18
	1, 2, 3, 4	64.57	1.37	8.15	80.47	0.13
S6	1	21.71	9.50	11.06	49.33	0.67
	1, 2	59.28	0.77	12.05	86.36	0.20
	1, 2, 3	58.02	1.96	8.97	77.69	0.16
	1, 2, 3, 4	62.75	1.51	6.61	77.12	0.11

## Data Availability

The data presented in this study are available on request from the corresponding author.

## Supplementary Materials

The following supporting information can be downloaded at: https://www.mdpi.com/article/10.3390/nano13030383/s1, Figure S1: Oscillator decomposition of S4 at 550 nm in six orders. Figure S2: Reflectance spectra with different Lorentz oscillator components for (a) S1, and the oscillator decomposition at a wavelength of (b) 250, (c) 300, (d) 325, (e) 375, (f) 600 and (g) 800 nm. Curves in (b)–(g) are the contour lines of reflectance. Figure S3: Reflectance spectra with different Lorentz oscillator components for (a) S3, and the oscillator decomposition at a wavelength of (b) 260, (c) 380, (d) 430, (e) 570, (f) 600 and (g) 800 nm. Curves in (b)–(g) are the contour lines of reflectance. Figure S4: Reflectance spectra with different Lorentz oscillator components for (a) S5, and the oscillator decomposition at a wavelength of (b) 270, (c) 340, (d) 400, (e) 585, (f) 700 and (g) 900 nm. Curves in (b)–(g) are the contour lines of reflectance.
