# Supplementary material for: Interpretation of Reflection and Colorimetry Characteristics of Indium-Particle Films by Means of Ellipsometric Modeling
Source: Nanomaterials (Basel). 2023 Jan 18;13(3):383. doi: 10.3390/nano13030383 (PMC9920837; doi:10.3390/nano13030383)
Supplement: Supplementary file 1 [file nanomaterials-13-00383-s001.zip › nanomaterials-2163030-supplementary.pdf]

Supplementary Materials:

Interpretation of Reflection and Colorimetry Characteristics of Indium-Particle Films by Means of Ellipsometric Modeling

Hao-Tian Zhang [1], Rong He [1], Lei Peng [1], Yu-Ting Yang [1], Xiao-Jie Sun [1], Yu-Shan Zhang [1], Yu-Xiang Zheng [1,2,*], Bao-Jian Liu [1,3], Rong-Jun Zhang [1], Song-You Wang [1], Jing Li [1], Young-Pak Lee [1,4] and Liang-Yao Chen [1]

[1] Department of Optical Science and Engineering, School of Information Science and Technology, Fudan University, Shanghai 200433, China
[2] High tech Center for new Materials, novel Devices and cutting-edge Manufacturing, Yiwu Research Institute of Fudan University, Yiwu 322000, China
[3] Shanghai Institute of Technical Physics, Chinese Academy of Sciences, Shanghai 200083, China
[4] Department of Physics, Quantum Photonic Science Research Center and RINS, Hanyang University, Seoul 04763, Republic of Korea
* Correspondence: yxzheng@fudan.edu.cn

1. Vector decomposition of S4 in different oscillator orders

For indium-particle film S4, the addition order of oscillators L2, L3 and L4 is changed at a wavelength of 550 nm (with the other components being fixed). Six decompositions are shown in Fig. S1. The effect of L2 maintains to be similar, regardless of the way of decomposition, as the longest vector behaves for a decreased n and an increased k. The vector of L3 is much shorter than that of L2. When L3 is located at a large n-k position, it induces a negative k, as analyzed in section 3.2.2 in the manuscript. When L3 is added before L2, the k comes to be larger, indicating that the extinction of light at this wavelength is enhanced by introducing L3. For L4, this results in increments for both n and k regardless of the addition order.

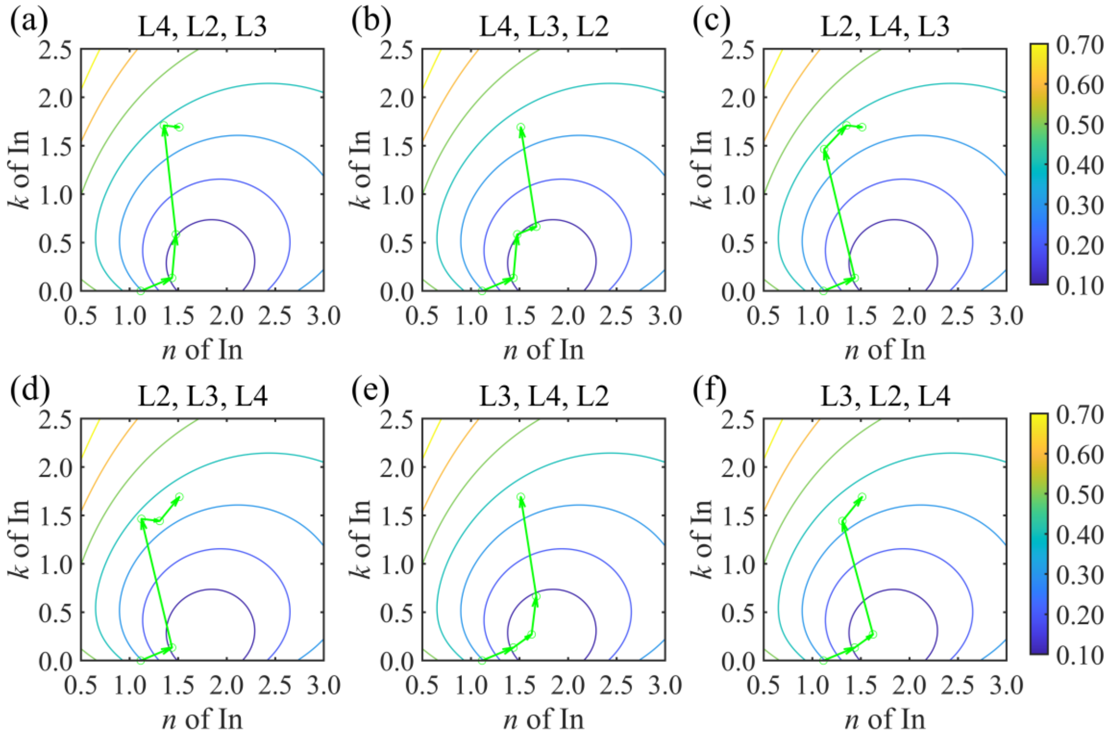

Figure S1. Oscillator decomposition of S4 at 550 nm in six orders

2. Oscillator decompositions for S1, S3 and S5

We also depict the oscillator decompositions for S1, S3 and S5, as shown in Figs. S2, S3 and S4, respectively.

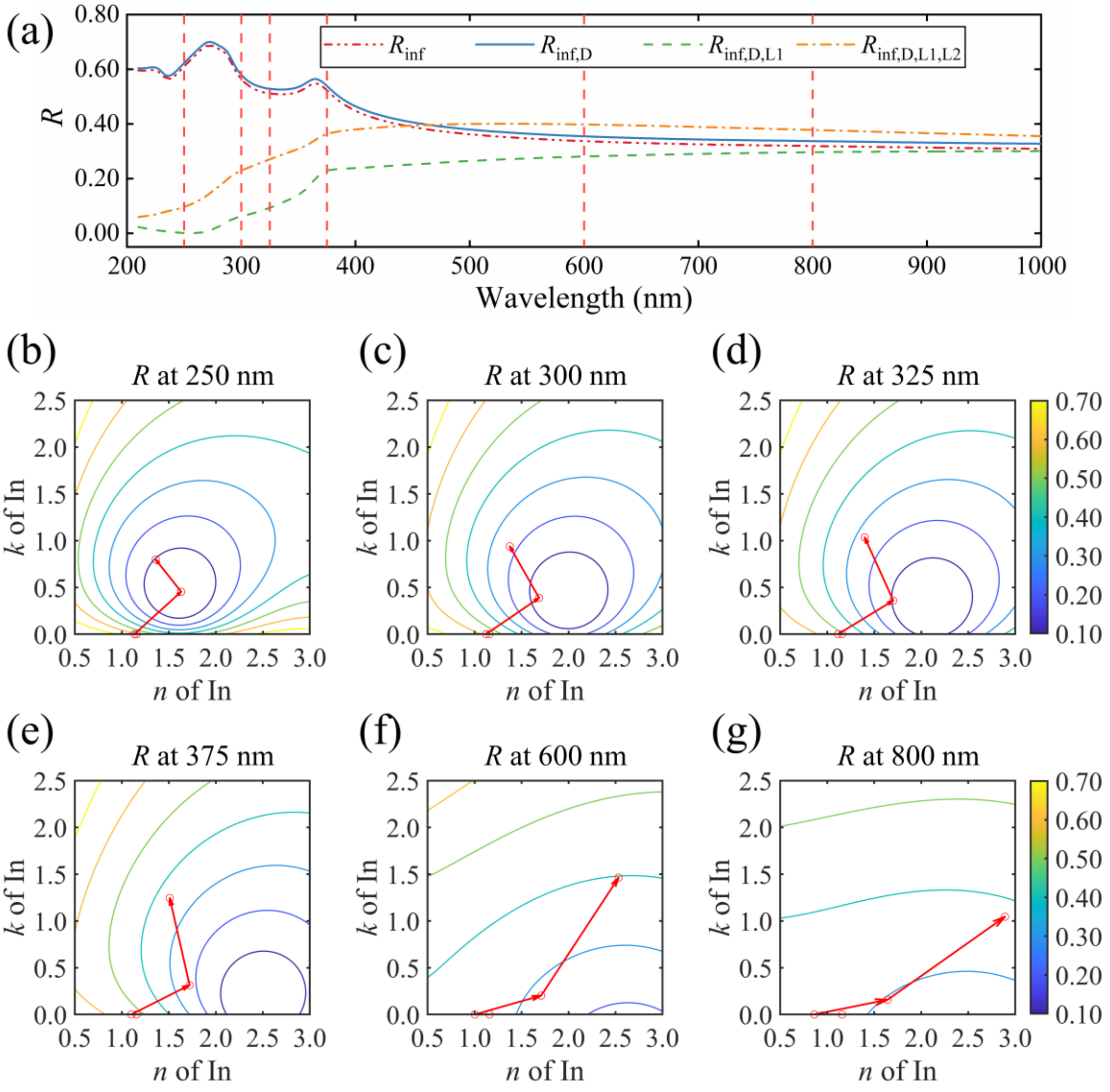

Figure S2. Reflectance spectra with different Lorentz-oscillator components for (a) S1, and the oscillator decomposition at a wavelength of (b) 250, (c) 300, (d) 325, (e) 375, (f) 600 and (g) 800 nm. Curves in (b)–(g) are the contour lines of reflectance.

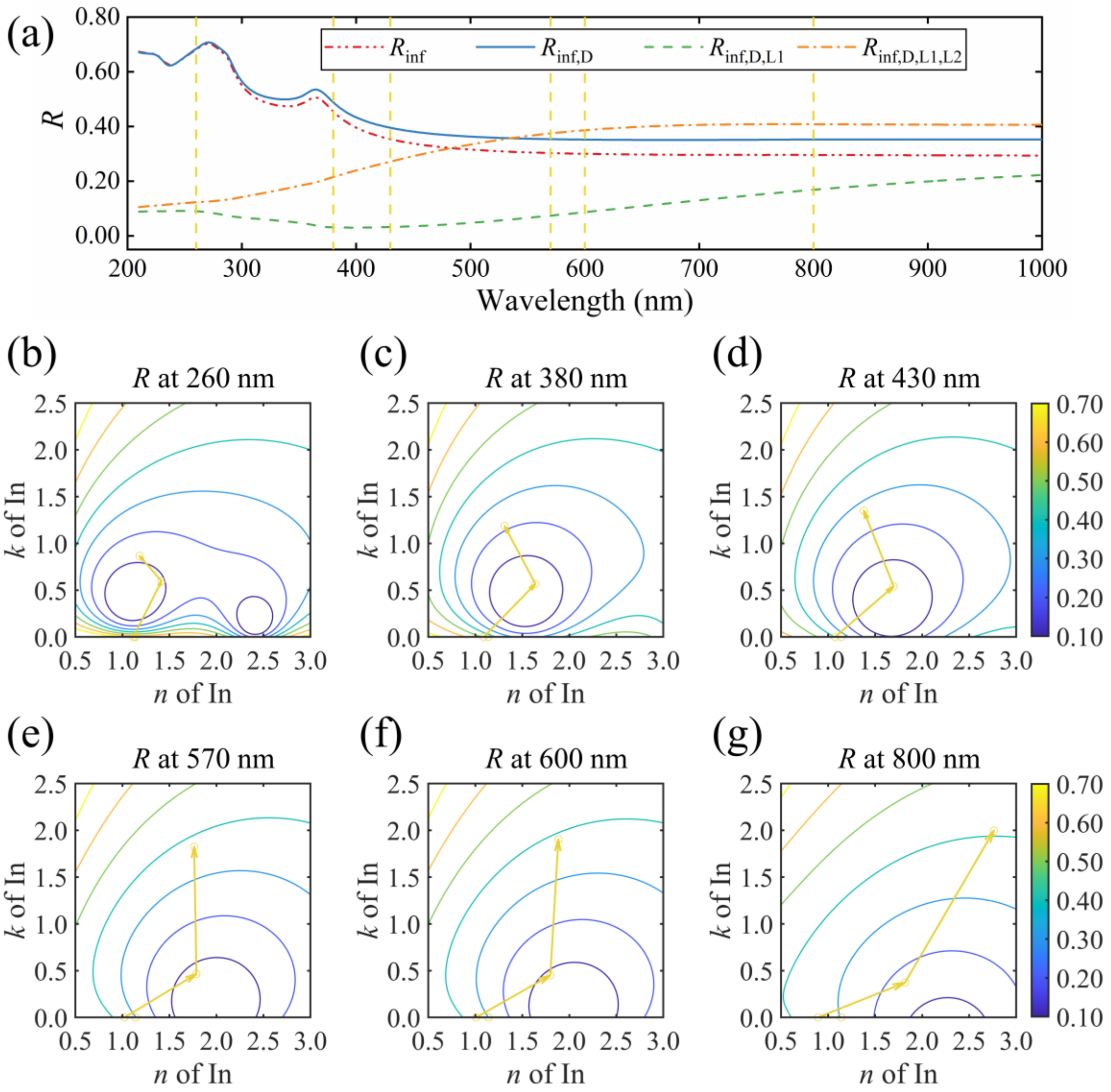

Figure S3. Reflectance spectra with different Lorentz-oscillator components for (a) S3, and the oscillator decomposition at a wavelength of (b) 260, (c) 380, (d) 430, (e) 570, (f) 600 and (g) 800 nm. Curves in (b)–(g) are the contour lines of reflectance.

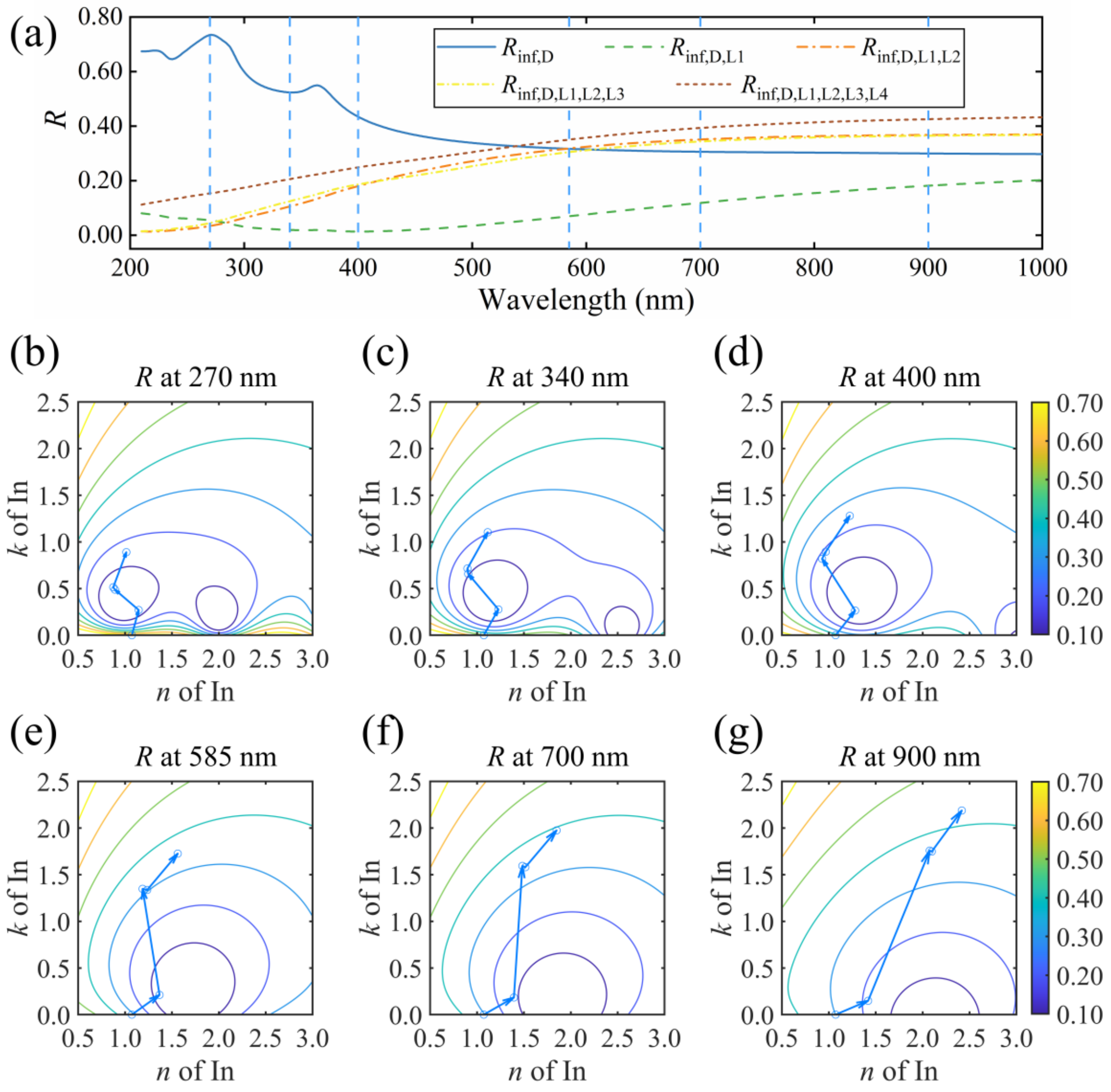

Figure S4. Reflectance spectra with different Lorentz-oscillator components for (a) S5, and the oscillator decomposition at a wavelength of (b) 270, (c) 340, (d) 400, (e) 585, (f) 700 and (g) 900 nm. Curves in (b)–(g) are the contour lines of reflectance.

3. Calculation of the colorimetric parameters

The calculation of colorimetric parameters was performed by converting the reflectance spectra in 380–780 nm into color, which is defined by the International Commission on illumination (CIE) [1]. The standard D65 illumination was used to calculate the reflected spectral intensity. The color-matching function of human eyes is related to the way we percept the spectral intensity. Parameters *X*, *Y* and *Z*, called the tristimulus values, corresponding to coordinates in the CIE 1931 color space, can be obtained by using the following equations:

$$X = \frac{\int_{380\ \text{nm}}^{780\ \text{nm}} I_{\text{D65}}(\lambda) R(\lambda) \bar{x}(\lambda) d\lambda}{\int_{380\ \text{nm}}^{780\ \text{nm}} I_{\text{D65}}(\lambda) \bar{y}(\lambda) d\lambda}, \tag{S1}$$

$$Y = \frac{\int_{380\text{ nm}}^{780\text{ nm}} I_{D65}(\lambda)R(\lambda)\bar{y}(\lambda)d\lambda}{\int_{380\text{ nm}}^{780\text{ nm}} I_{D65}(\lambda)\bar{y}(\lambda)d\lambda}, \quad \text{(S2)}$$

$$Z = \frac{\int_{380\text{ nm}}^{780\text{ nm}} I_{D65}(\lambda)R(\lambda)\bar{z}(\lambda)d\lambda}{\int_{380\text{ nm}}^{780\text{ nm}} I_{D65}(\lambda)\bar{y}(\lambda)d\lambda}, \quad \text{(S3)}$$

where $R(\lambda)$ is the reflectance spectrum of sample, $I_{D65}(\lambda)$ is the spectral irradiance of standard D65 source, $\bar{x}(\lambda)$, $\bar{y}(\lambda)$ and $\bar{z}(\lambda)$ are the color matching functions of human eyes. To eliminate the influence of light source, the tristimulus values are further transformed into the $L^*a^*b^*$ color coordinates by using Eqs. (S4)–(S7):

$$L^* = 116f(Y/Y_n) - 16, \quad \text{(S4)}$$

$$a^* = 500[f(X/X_n) - f(Y/Y_n)], \quad \text{(S5)}$$

$$b^* = 200[f(Y/Y_n) - f(Z/Z_n)], \quad \text{(S6)}$$

$$f(q) = \begin{cases} q^{1/3} & q > (\frac{6}{29})^3 \\ \frac{q}{3\times(\frac{6}{29})^2} - 16/116 & q \le (\frac{6}{29})^3 \end{cases}, \quad \text{(S7)}$$

where X, Y and Z are the tristimulus values, and X_n, Y_n and Z_n are the white point ones of standard illuminant. L^* describes the lightness of color. Furthermore, chroma $C=(a^{*2}+b^{*2})^{1/2}$ and hue $h=\arctan(b^*/a^*)$ are calculated. Eventually, saturation S is evaluated by using $S=C/L^*$.

Reference

1. Schanda, J. CIE Colorimetry. In *Colorimetry: Understanding the CIE System*, Schanda, J., John Wiley & Sons: Hoboken, United States, 2007; Volume 3, pp. 25–78.
